# Temperature and humidity limits for flight activity of field-collected *Culicoides* biting midges (Diptera: Ceratopogonidae) in the United Kingdom under defined laboratory conditions

**DOI:** 10.1093/jme/tjag058

**Published:** 2026-04-23

**Authors:** Laura A Jones, Simon Gubbins, Christopher J Sanders

**Affiliations:** The Pirbright Institute, Woking, UK; The Pirbright Institute, Woking, UK; The Pirbright Institute, Woking, UK

**Keywords:** *Culicoides*, temperature, humidity, flight, dispersal

## Abstract

*Culicoides* biting midges are biological vectors of internationally important arboviruses, including bluetongue virus (BTV) and epizootic hemorrhagic disease virus (EHDV), which significantly impact livestock production in Europe. Temperature is a fundamental driver of the transmission of *Culicoides*-borne arboviruses, regulating virus replication within the insect, vector survival and activity. A key determinant of the spread of *Culicoides*-borne viruses is vector dispersal through flight activity which is constrained by temperature. Here we determine the temperature and humidity thresholds of flight activity for UK *Culicoides* vector species.

More than 40,000 adult *Culicoides* were collected from field sites in the southeast of England using CDC light traps during spring, summer, and autumn. Bioassays conducted under laboratory conditions were used to determine temperature and humidity thresholds of *Culicoides* flight activity by measuring flight phototaxis towards an ultraviolet light source over a 24-h period.

The highest flight activity (48%) was observed at temperatures between 20 and 25 °C, with reduced activity above and below this. Flight activity at 10 and 35 °C was reduced, with less than 10% of *Culicoides* active. Moreover, seasonal variation in low and high temperature thresholds for flight were recorded, with a lower threshold for flight activity recorded in populations caught in spring compared with those caught in summer and autumn. Finally, *Culicoides* flight activity and survival was significantly reduced under low humidity conditions (<50% rH).

The flight response of UK *Culicoides* vector species to temperature and humidity presented here will facilitate the refinement of existing models used to predict incursion and spread of *Culicoides*-borne viruses.

## Introduction

*Culicoides* biting midges transmit important arboviruses affecting livestock and wild mammals including bluetongue virus (BTV), epizootic hemorrhagic disease virus (EHDV) and Schmallenberg virus (SBV). Over the last two decades, the distribution of *Culicoides*-borne viruses has expanded, with new serotypes and strains emerging and spreading across Europe causing significant economic losses (Velthuis et al. 2010, Gethmann et al. 2020). Since 2006, northern Europe has experienced multiple outbreaks of different BTV serotypes and strains, the most recent being the emergence and spread of BTV-3 since 2023 (Holwerda et al. 2024). Similarly, EHDV-8 emerged in Sardinia in 2022, subsequently spreading into mainland Europe for the first time, where clinical disease in cattle has been observed (Martinez et al. 2025).

Meteorological conditions determine the ecology and behavior of *Culicoides* vectors, influencing the epidemiology of the pathogens they transmit (Wittmann and Baylis 2000). Temperature plays a key role in the transmission and persistence of *Culicoides*-borne arboviruses, affecting both the rate of replication and dissemination of an arbovirus within its biological vector (Wittmann et al. 2002, Carpenter et al. 2011) and *Culicoides* population dynamics and activity (Sanders et al. 2011, Elbers et al. 2015, Sanders et al. 2019, Hudson et al. 2023). Mark-recapture techniques have previously determined that *Culicoides* can actively disperse a few kilometers from their breeding habitats (Sanders et al. 2017). Yet, due to their small body size, virus incursion evidence suggests that long distance wind-borne dispersal of *Culicoides* can occur, facilitating the spread of *Culicoides-*borne diseases over long distances (Chapman et al. 2004, Gloster et al. 2008). Indeed, wind-borne incursion of infected *Culicoides* has been identified as the most likely route of incursion to the UK of BTV-8 in 2007 (Gloster et al. 2008, Burgin et al. 2013) and BTV-3 in 2023 (Defra 2024).

Modelling of the probability of dispersal of *Culicoides* forms a key component of the risk assessment of incursion of *Culicoides*-borne viruses to the UK (Defra 2024). Since the 2006 to 2008 BTV-8 outbreak, the UK Met Office numerical atmospheric-dispersion modelling environment (NAME) has been used to model and predict risk of wind-borne incursion of infected *Culicoides* from northern Europe according to meteorological conditions (Burgin et al. 2013). Such models rely on empirical data of *Culicoides* flight activity under different meteorological conditions (Sanders et al. 2011, Sanders et al. 2012, Burgin et al. 2013).

Previous studies have measured the effect of temperature on *Culicoides* flight activity, in response to light, under laboratory conditions (Tsutsui et al. 2011, Venter et al. 2019, Tugwell et al. 2021). In general, lower temperatures were associated with reduced flight activity, although the temperature at which activity ceased appears to be species-specific (Tsutsui et al. 2011, Tugwell et al. 2021). Activity of *Culicoides imicola* (Kieffer) was significantly decreased at temperatures below 10 °C (Venter et al. 2019). This was similar to activity of *Culicoides* in the subgenus *Avaritia* (Fox) in the south of England, where less than 5% of the population was active at temperatures below 10 °C and 4 °C for summer and autumn populations respectively (Tugwell et al. 2021). In contrast, collections in the north of England, where *Culicoides impunctatus* (Goetghebuer) was the predominant species, revealed these populations did not become active until temperatures reached 14 °C (Tugwell et al. 2021). Similarly, studies of *Culicoides oxystoma* (Kieffer) and *Culicoides maculatus* (Shiraki) from Japan showed that active flight was recorded in less than 10% of individuals when temperatures dropped below 16 to 17 °C, although very low levels of activity were still recorded at 6 °C (Tsutsui et al. 2011). Response to higher temperatures was again shown to be dependent on species and region, with one study reporting high activity levels of *C. oxystoma* at temperatures above 25 °C (Tsutsui et al. 2011) whilst another study reported a suppression of activity of *C. imicola* above 20 °C which remained stable up to 30 °C (Venter et al. 2019).

Relative humidity (rH) also plays a key role in shaping ecology and behavior of insects, inversely interacting with temperature to influence insect fitness. Given their small body size, *Culicoides* are acutely sensitive to desiccation and changes to humidity, seeking out optimal microclimates to regulate their water balance (Purse et al. 2015). Significant fitness costs can occur when organisms become dehydrated or overhydrated (Chown and Nicolson 2004, Brown et al. 2023), with insects able to regulate water balance through a number of physiological and behavioral adaptations including changes in activity level (Hagan et al. 2018). Although a previous study did not identify relative humidity as a key factor influencing activity in *C. imicola* under laboratory conditions (Venter et al. 2019), studies on mosquitoes have demonstrated that relative humidity influences activity positively (Rudolfs 1925, Drakou et al. 2020), with a significant decline in activity at low humidity (Thomson 1938, Grimstad and DeFoliart 1975, Hagan et al. 2018).

Age structure is an important indicator of potential vector status of *Culicoides*, with older females identified through the presence of abdominal pigmentation following completion of the first gonotrophic cycle (Dyce 1969). Pigmented females are most epidemiologically relevant as having already acquired at least one bloodmeal, are the portion of the population most likely to carry a transmissible infection. Previous work has demonstrated that *Culicoides* of different physiological states and sexes can be differentially drawn to various attractants (Anderson and Linhares 1989, Gerry and Mullens 1998, Mullens and Gerry 1998, Venter et al. 2009, Harrup et al. 2012). Differences in timing of onset and peak host-seeking between pigmented and unpigmented females of *C. sonorensis* has also been reported (Zhang et al. 2024). It is therefore possible that flight activity and survival in response to different temperatures and humidities may vary between *Culicoides* of different physiological status. Whilst previous work did not identify differences in flight activity according to physiological status (Tugwell et al. 2021), most studies investigating the effect of temperature on flight activity of *Culicoides* under laboratory conditions does not separate response by physiological status.

Previous studies in the UK have contributed to the development and refinement of modelling *Culicoides* flight in NAME. One study determined the low temperature threshold for Palearctic *Culicoides* flight activity at 4 °C (Burgin et al. 2013, Tugwell et al. 2021). However, high temperature thresholds for activity were not investigated as temperatures above 14 °C were not evaluated and the potential impact of relative humidity on active flight was not addressed (Tugwell et al. 2021). Importantly, the study observed that the season in which vector populations have developed may lead to variation in response to climatic conditions by vector populations, altering the degree of risk with time of incursion (Tugwell et al. 2021).

This study aims to further parameterize the response of *Culicoides* to meteorological conditions. Flight activity of UK *Culicoides* was assessed in the laboratory across an expanded temperature range reflective of the temperatures experienced in the European summer. Upper temperature thresholds of activity at timepoints across the vector season were determined. Further, the impact of relative humidity on the initiation of *Culicoides* flight was investigated. The effects of temperature and humidity on adult *Culicoides* survival at each timepoint were also assessed. These data can be used to define *Culicoides* activity for further refinement of models used to predict potential incursions and spread of infected *Culicoides.*

## Materials and Methods

### Field Collection of Adult *Culicoides*

**Fig. 1.**
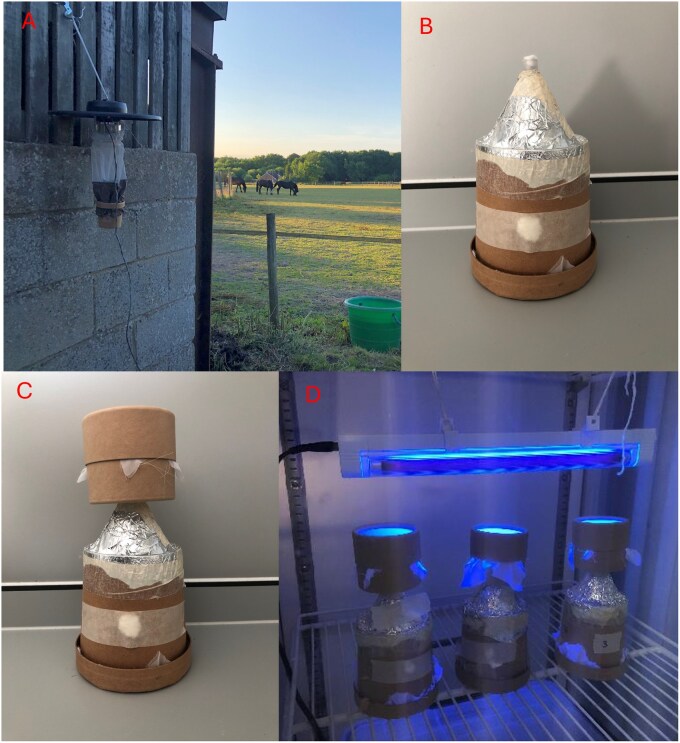
Equipment used for collecting and analyzing *Culicoides* for flight activity and survival at different temperatures and humidity levels. Cardboard collection pots (12 oz) containing sucrose-soaked cotton wool pads were attached to CDC light suction traps for live collection of *Culicoides* (A). Insects were sorted into assay pots (B) and acclimatized for 2 h at desired temperature and humidity conditions before a collection pot was placed on top (C) and placed under an ultraviolet light for 24 h (D).

Field collections of adult *Culicoides* were conducted across three equine holdings in the south of England at different times of the *Culicoides* activity period. Collections were conducted to represent early (May to June), mid- (July to August) and late (September to October) timepoints in the vector season in 2022 and 2023. Collections were conducted over a two-week period at each timepoint to account for generational differences in response. Down-drought miniature blacklight (UV) Centers for Disease Control (CDC) light traps (model 912, John W Hock Co, Florida, United States) were used to collect host-seeking adult *Culicoides.* Traps were suspended at a height of approximately 1.5 m above ground. Traps were set up at least two hours prior to sunset and run overnight before collection of live insects within 2 h of sunrise the following morning. Insects were collected live into 12 oz cardboard pots (Cater4u, United Kingdom) containing a cotton pad soaked in 10% sucrose to provide a sugar source and promote survival within the trap. Cardboard collection pots were secured to the CDC trap using a CDC sleeve and elastic bands (Fig. 1A). Surplus insects not used in flight assays were not identified to species level and survival within the trap was not estimated.

### Flight Activity Experiments

Collected insects were transferred to the Pirbright Institute for flight activity experiments. Insects collected in a single night’s trapping at the three sites were pooled and released into cages (MegaView Science Co. Ltd., Taiwan). From these cages, approximately 100 to 150 adult *Culicoides* were allocated to each flight assay chamber using an electric pooter for each trial. Flight assay chambers were adapted from those previously described (Tugwell et al. 2021). They consisted of a 12 oz carboard pot with the bottom removed and a translucent funnel (66 mm top diameter; 7.4 cm in length) attached. The funnel was covered in aluminium foil to prevent light penetration into the chamber (Fig. 1B). A fine mesh was used to cover the bottom of the chamber. Cotton wool was used to plug the top of the funnel to prevent premature escape of *Culicoides* from the assay chamber.

Initial activity trials tested a total of six temperatures (10, 15, 20, 25, 30, and 35 °C) at a consistent humidity of 75% rH and five humidities (35, 50, 60, 80, 90% rH) at a consistent 20 °C. Results from these trials were used to select temperatures and humidities to investigate temperature and humidity interactions (temperatures: 15, 25, 30 °C and humidities: 35, 50, 90% rH). For all temperature and humidity combinations, apart from the 35% rH groups, a temperature and humidity-controlled incubator (Panasonic, United Kingdom) was used to maintain conditions for the duration of the experiment. For groups requiring 35% rH, a container of saturated potassium acetate (Sigma, United Kingdom) solution was added to the temperature-controlled incubator (Panasonic, United Kingdom) to maintain 35% rH.

Once filled with *Culicoides*, flight assay chambers were randomly allocated to a test temperature and humidity combination and placed into an incubator and left to acclimatize for 2 h in darkness prior to the start of the experiment as previously described (Tugwell et al. 2021). Following acclimatization, the cotton wool plugging the top of the funnel was removed and replaced with a collection pot (Watkins and Doncaster) and placed under a UV light (Fig. 1C and D) which was active for the 24-h period. Assay pots were placed inside a tray to maintain darkness within the pot so that the UV light above the collection pot was the only light source within the incubator. Both collection pots and assay chambers were provided with cotton pads soaked in 10% sucrose to maintain insects. Assay chambers were left with continuous UV illumination for 24 h to account for circadian, crepuscular activity cycles, following which the top collection pot was detached from the original flight assay chambers and both sealed with cotton wool and placed at −20 °C, as previously described (Tugwell et al. 2021). The funnel design and material within the flight assay chamber aimed to reduce the possibility of *Culicoides* walking into the collection pot, although direct observations were not made. Upward movement into the collection pot was assumed to represent active flight of *Culicoides* and that the funnel design would prevent return to the lower chamber. Assays pots consisted of insects collected from a single trapping event for each temperature/humidity combination.

*Culicoides* flight activity was determined by calculating the number of *Culicoides* moving into the top collection pot compared with the number of total *Culicoides* remaining in the original flight assay chamber. Thermal threshold for activity was defined as the temperature at which <5% of the population was active, as previously described (Tugwell et al. 2021). *Culicoides* survival was determined by observing the number of *Culicoides* alive at the time of freezing. *Culicoides*, which were dead at the time of freezing, were identified through their desiccated appearance.

### Sample Identification

*Culicoides* were morphologically identified to species level by wing patterns under a dissecting microscope (Leica Microsystems, Germany) using published keys (Campbell and Pelham-Clinton 1960, Boorman 1986, Mathieu et al. 2012). Adult *Culicoides* were grouped into four categories: *C. obsoletus* group, *Culicoides pulicaris* group (Linnaeus)*, Culicoides achrayi* (Kettle and Lawson) and other *Culicoides*. Female *Culicoides* were also identified to physiological state by examination of the abdomen and assigned to one of the following categories: unpigmented, pigmented, gravid and blood-fed (Dyce 1969).

A sub-sample of 40 females (20 flying, 20 not flying) within the *C. obsoletus* group from each cohort were identified further to species level (*C*. *obsoletus* (Meigen) and *C. scoticus* (Downes and Kettle) only) using an adapted multiplex PCR method targeting the internal transcribed spacer (ITS) region (Mathieu et al. 2011, Tugwell et al. 2021).

*Culicoides* were transferred to individual collection tubes with 200 µl tissue digest solution containing 100 mM Tris-HCL (pH 8.0) (Invitrogen by Thermo Fisher, United Kingdom), 200 mM NaCl (Invitrogen, United Kingdom), 0.2% (w/v) SDS (Merck Life Science UK Limited, United Kingdom) 5 mM UltraPure EDTA (pH 8.0) (Invitrogen, United Kingdom), 200 µg/ml proteinase K (Qiagen, United Kingdom) and made up to a total volume of 200 µl per sample using nuclease-free water (Invitrogen, United Kingdom). Following an overnight incubation at 37 °C, individual *Culicoides* specimens were transferred to tubes containing 70% ethanol for storage. *Culicoides* DNA was then extracted from 100 µl of tissue digest solution and eluted into 100 µl buffer using the Kingfisher Flex automated extraction platform and the MagMAX CORE Nucleic Acid Purification Kit according to the manufacturer’s instructions.

Amplification of the ITS region was performed in a total volume of 10 µl consisting of 1× TaqMan Fast Advanced MasterMix (Thermo Fisher, United Kingdom), 0.3 µM of each primer, 0.2 µM of each probe, 2 µl DNA. The thermal profile consisted of an initial cycle of 50 °C for 2 min followed by 95 °C for 2 min, followed by 40 cycles of 95°C for 3 s, and 60 °C for 30 s.

### Statistical Modelling

A generalized linear mixed model (GLMMs) was used to investigate the relationship between temperature, relative humidity and the proportion of *Culicoides* flying and how this relationship differed amongst spring, summer and autumn cohorts. More precisely, a binomial family GLMM with logit link function was constructed with the proportion of *Culicoides* flying, *p*, as the response variable. The initial model included temperature (*T*) and relative humidity (*H*) as linear and quadratic terms, so that,


$$\log(\frac{p}{1-p})=\sum_{i=0}^{2}\sum_{j=0}^{2}b_{ij}T^{i}H^{j}$$

where the *b_ij_*s are allowed to differ amongst cohorts (ie spring, summer, and autumn). In addition, pot was included as a random effect (to allow for between-pot variation). Model selection proceeded by stepwise deletion of non-significant (*P *> 0.05) terms as judged by likelihood ratio tests.

To assess whether there were differences in flight activity between pigmented and unpigmented individuals, two models were compared using a likelihood ratio test. The first was the best fitting GLMM from the preceding analysis and the second was the best fitting GLMM but with all model parameters allowed to differ between pigmented and unpigmented individuals.

The relationship between survival, temperature and relative humidity was analyzed using the same approach as described above, except the proportion of flying midges which survived was the response variable. When considering survival, the effects of pigmentation status and differences between *C. obsoletus* and *C. scoticus* were not examined.

All models were implemented using the lme4 package (Bates et al. 2015) in R (version 4.4.0) (R Core Team 2024). To improve convergence during model fitting, temperature and relative humidity were centred on their means and scaled by their standard deviations.

## Results

A total of 41,936 *Culicoides* were collected between May 2022 and October 2023 (Table 1). All collections were dominated by the *C. obsoletus* group (78.85%) with the remaining insects identified as *C. pulicaris* (7.22%), *C. achrayi* (11.45%), and other *Culicoides* (2.45%). *Culicoides* were most frequently classified as unpigmented (58.38%), and pigmented (32.23%). Only small numbers of males and gravid or blood-fed females were collected, so no further analyses were conducted on these individuals.

**Table 1.** Numbers of adult *Culicoides* used in each cohort and the percentage (%) of species and pigmentation status

		Cohort		
		Spring	Summer	Autumn
**Total number of *Culicoides* tested**		22,349	13,390	6,197
**Species by season**	*C. obsoletus* group	16,275 (72.82%)	11,532 (86.12%)	5,291 (85.38%)
	*C. pulicaris* group	1,610 (7.20%)	858 (6.41%)	556 (8.97%)
	*C. achrayi*	3,998 (17.89%)	694 (5.18%)	95 (1.53%)
	Other *Culicoides*	466 (2.09%)	306 (2.29%)	255 (4.11%)
**Pigmentation by season**	Pigmented	7,620 (34.10%)	4,281 (31.97%)	1,615 (26.06%)
	Unpigmented	12,625 (56.49%)	7,797 (58.23%)	4,069 (65.66%)
	Gravid	1,904 (8.52%)	1,011 (7.55%)	372 (6.00%)
	Blood-fed	98 (0.44%)	73 (0.55%)	60 (0.97%)
	Male	102 (0.46%)	228 (1.70%)	81 (1.31%)

Within the *C. obsoletus* group, a significant change in proportion of individuals identified as *C. obsoletus* and *C. scoticus* was seen between the seasons (χ^2^ = 8.8, df = 2, *P *= 0.012). *Culicoides scoticus* was the predominant species identified early in the season, making up 75% of the population tested, compared with only 44.8% of the population late in the season.

### Flight Activity

**Fig. 2.**
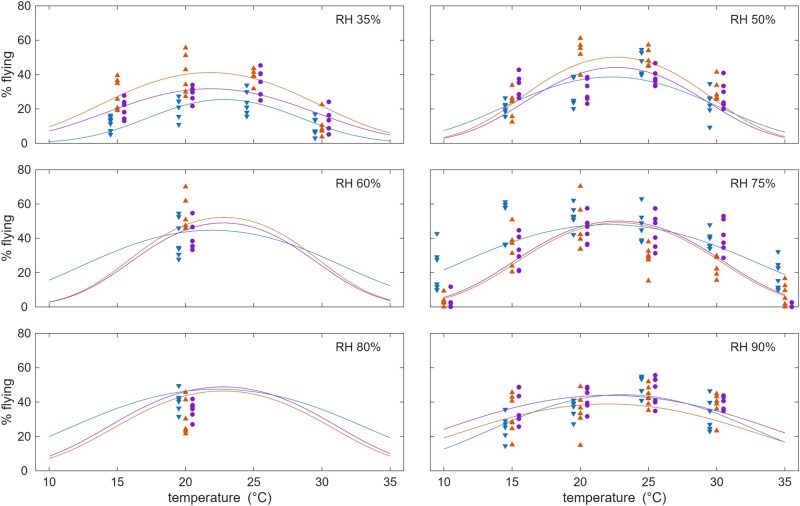
The impact of temperature and humidity on *Culicoides* flight activity. Each plot shows the observed (symbols) and expected proportion of *Culicoides* flying towards ultraviolet light at different temperatures and relative humidities. Color indicates cohort: spring (blue), summer (orange), and autumn (purple).

Temperature and humidity were shown to significantly influence flight activity of adult *Culicoides* (Fig. 2; Supplementary Table S1). The percentage of the population actively flying towards a UV light ranged from 8.7% to 47.9% (*n* = 1,066 and 1,587 tested respectively) under the different temperatures and humidities tested. Across all cohorts, the highest *Culicoides* flight activity (47.9%) was observed at a temperature of 20 °C and 75% relative humidity. The proportion of active *Culicoides* decreased with temperatures above and below this point (Fig. 2). At the extremes of the temperature range tested (10 and 35 °C), less than 10% of *Culicoides* were found to be active across all cohorts. *Culicoides* flight activity was highest at humidities between 50% and 80% but was reduced at lower and higher humidities.

The time of year when *Culicoides* were collected influenced the minimum temperature at which they were actively flying, and the levels of activity reached at the temperature extremes tested (Fig. 2). *Culicoides* collected early in the season were shown to have increased activity at the high and low temperature extremes tested compared with collections from the middle and end of the season. Despite this, similar levels of activity were reached at intermediate temperatures (20 to 25 °C). Individuals collected early in the season were shown to be significantly more active in the 10 °C group (22.4%) compared with those collected in the middle (3.6%) and the end (3.2%) of the season. A similar pattern was observed at higher temperatures, with a reduction in activity of *Culicoides* tested at 35 °C collected in the middle (7.6%) and the end (0.45%) of the season compared with those collected at the beginning (18.2%) of the season.

**Fig. 3.**
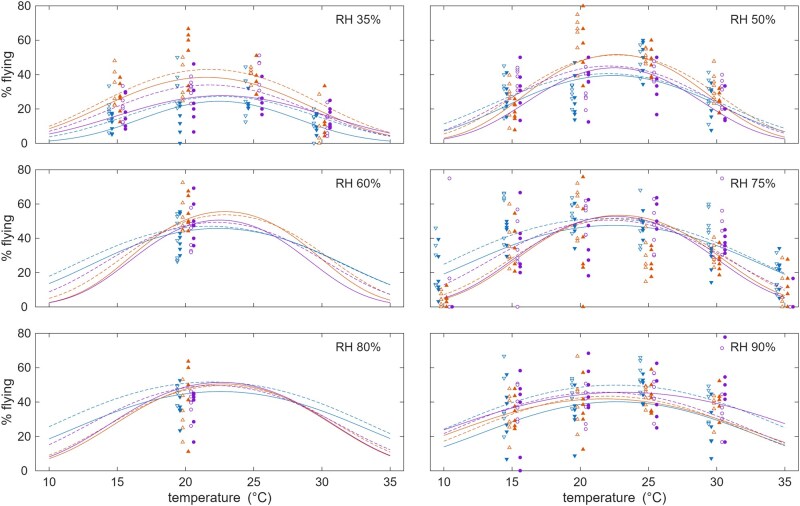
The effect of temperature and humidity on *Culicoides* flight activity according to pigmentation status. Each plot shows the observed (symbols) and expected proportion of *Culicoides* flying towards ultraviolet light at different temperatures and relative humidities. Color indicates cohort: spring (blue), summer (orange), and autumn (purple), while line style/symbol fill shows pigmentation: unpigmented (dashed line or open symbol) or pigmented (solid line or filled symbol).

A significant difference was also seen between flight activity of pigmented compared with unpigmented females (*P *< 0.001) (Fig. 3). Pigmented females were less likely to be active at all temperatures and humidities tested, compared with unpigmented females.

### Survival

**Fig. 4.**
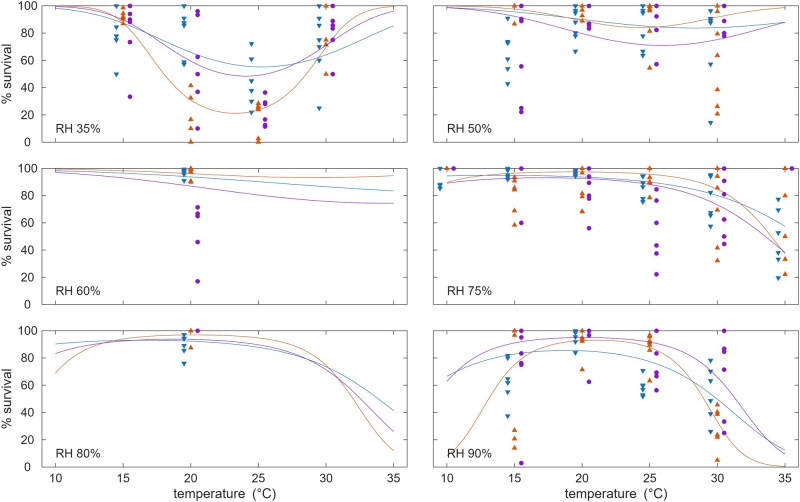
The effects of temperature and humidity on adult *Culicoides* survival over a 24-h period. Each plot shows the observed (symbols) and expected proportion of *Culicoides* surviving at different temperatures and relative humidities. Plot color indicates cohort: spring (blue), summer (orange), and autumn (purple).

Survival of *Culicoides* over a 24-h period was also shown to be impacted by both temperature and humidity (Fig. 4; Supplementary Table S2). At lower humidities (<50%), higher levels of survival were observed at lower (<15 °C) and higher temperatures (>30 °C) than at intermediate temperatures (15 to 25 °C). By contrast, at higher humidities (>50%) survival did not vary greatly with temperature, except at the highest temperatures (>30 °C) where it decreased markedly. Survival differed amongst *Culicoides* caught at different times of the season (Supplementary Table S2), but these differences lacked a consistent direction across temperatures or humidities (Fig. 4).

## Discussion

Many physiological processes in insects are temperature-dependent, therefore understanding thermal limits are important for determining the activity and survival of *Culicoides* during extreme conditions. This study has demonstrated that both temperature and humidity influence the flight activity and survival of UK *Culicoides*, identifying differential responses according to season under laboratory conditions. Whilst flight activity and survival at intermediate temperatures and high humidity conditions were consistent, *Culicoides* collected earlier in the season were shown to have higher tolerance for the high and low temperatures tested during this study compared with individuals collected later in the season.

In northern Europe, the number of *Culicoides* generations occurring each year remains unclear, yet previous research has suggested bi- or tri-voltinism for populations of *C. impunctatus* (Blackwell et al. 1992) and the subgenus *Avaritia* (Birley and Boorman 1982, Stokes et al. 2022). Results presented here highlight significant differences in thermal tolerance of populations presumed to be from distinct generations. *Culicoides* collected early in the season were shown to have higher activity levels and survival rates at the high and low temperature extremes tested within the current study compared with individuals collected from the middle or end of the season. Likewise, higher activity and survival were associated with early season collections at low humidity conditions (<50% rH) compared with later collections. Differences in lower thermal threshold for flight have also been identified in the subgenus *Avaritia* in the south of England. Interestingly, these results showed that populations collected in the autumn were active at lower temperatures compared with those collected in the summer (Tugwell et al. 2021), contrary to our findings. Some studies have documented the influence of larval habitat parameters, including temperature, on resulting adult traits, suggesting a possible mechanism underlying the differential responses observed between cohorts. It is also possible that seasonal differences in activity levels at the temperature and humidity extremes could reflect changes in species composition. Future work should aim to understand the drivers of variation in behavior, biology and ecology between generations of *Culicoides*, how this may be influenced by the larval habitats in which they develop and the potential variation between years.

Flight activity of *Culicoides* was closely linked to temperature, with a reduction in activity at the low and high temperature extremes tested within this study. *Culicoides* activity increased as temperatures rose between 10 and 20 °C, similar to previous findings (Tsutsui et al. 2011, Venter et al. 2019, Tugwell et al. 2021). Activity was reduced at temperatures above 20 °C, with a significant suppression in activity in the 35 °C group. Previous studies have presented equivocal results for flight activity at temperatures exceeding 20 °C with one study noting a continued increase in activity until approximately 25 °C, after which the proportion of *Culicoides* flying plateaued (Tsutsui et al. 2011). In contrast, at temperatures above 20 °C activity levels of *C. imicola* declined (Venter et al. 2019), similar to the current study, potentially indicating a species-specific response. Equally, field studies have obtained similar results for members of the *Avaritia* subgenus, with a reduction in trap collections when temperatures exceeded 21 °C in the UK (Sanders et al. 2012), whilst a delay in activity period of *C. imicola* was identified when average nighttime temperature rose above 19 °C (Venter et al. 2019). Collection of adult *Culicoides* from sheep using drop-traps have also suggested an optimal temperature of biting activity between 20 and 22 °C (Carpenter et al. 2008). Prior short-term exposure to low or high temperatures has been shown to improve tolerance of *Culicoides* in extreme temperatures (Nunamaker 1993, Verhoef et al. 2014), it would therefore be interesting to investigate the potential for acclimatisation to alter flight activity of *Culicoides* under warming climate conditions in the future.

Relative humidity was also shown to influence *Culicoides* activity with low humidity conditions (<50% rH) associated with lower activity levels in adult *Culicoides* across all cohorts. In contrast, a previous study found no correlation between relative humidity and flight activity of *C. imicola* under laboratory conditions, hypothesizing that humidity may play a secondary role in flight initiation (Venter et al. 2019). However, differences in experimental design, including the humidity ranges tested, may limit direct comparison between the studies.

Due to their small body size, *Culicoides* are acutely sensitive to desiccation, and it is likely that survival is influenced by both temperature and humidity. Indeed, our results revealed reduced survival of *Culicoides* at high temperature and low humidity conditions. Increasing temperatures have previously been linked to reduced survival in colony *C. sonorensis* (Wittmann et al. 2002, Rozo-Lopez and Drolet 2024), however, humidity alone did not impact survival (Wittmann et al. 2002). Vector longevity is a major determinant of vectorial capacity, and previous work has shown that intermediate temperatures can optimize virus transmission by maximizing survival whilst maintaining optimal ranges for viral replication (Rozo-Lopez and Drolet 2024). Indeed, vector survival was shown to be greatest under conditions favorable for transmission. Determining the longevity of *Culicoides* under these constant conditions as well as fluctuating conditions experienced under natural field settings would be beneficial.

The *C. obsoletus* group were the predominant species identified across all collections, yet the relative abundance of *C. scoticus* and *C. obsoletus* varied between the seasons. Differential abundances of these cryptic species have previously been identified from populations collected in the south of England (Tugwell et al. 2021) and Germany (Balczun et al. 2009), although the seasonal patterns of the two species differed between studies. Moreover, previous work has shown that *C. scoticus* are significantly more active compared with *C. obsoletus* (Tugwell et al. 2021). Whilst results presented here indicate a similar pattern of increased activity of *C. scoticus* across all cohorts, testing of a larger subset of individuals would be required to confirm this. It is possible that seasonal differences in activity levels at the temperature and humidity extremes recorded in this study may, in part, reflect the changes in species composition of the *C. obsoletus* group throughout the season. Although differences in behavior and vectorial capacity of the two cryptic species have yet to be fully explored, variation in seasonality and activity between *C. obsoletus* and *C. scoticus* could have implications for disease transmission. This could be particularly important when defining the highest transmission risk period, which could vary between the two species.

Behavioral disparity between females of different age and physiological status have previously been reported, including changes to timing of host-seeking (Zhang et al. 2024). Other studies have also shown differences in abundance in trap catches based on trapping method (Viennet et al. 2011), attractant used (McDermott et al. 2016) or collection site (Gerry and Mullens 2000, McDermott et al. 2016) further suggesting behavioral differences between the two. In this study, unpigmented females were found to be significantly more active compared with pigmented individuals under laboratory conditions, contrary to previous findings (Tugwell et al. 2021). The higher activity level of unpigmented compared with pigmented *Culicoides* may indicate that physiological status could influence activity level of *Culicoides*. Alternatively, the increased activity noted within this study may be due to difference in attraction to UV light between pigmented and unpigmented females although additional work would be required to investigate this further.

In the present study, phototaxic flight of *Culicoides* is assumed to have taken placed within the assay chambers. Direct observation of the flight behavior of individual *Culicoides* according to physiological and infection status in the laboratory would eliminate the need for this assumption, however, the size of *Culicoides* makes such observation technically challenging. Data presented here provide insights into the effects of temperature and humidity on *Culicoides* flight activity, which will be incorporated into the NAME dispersion model. However, several other factors have also been shown to influence flight activity and survival of *Culicoides*, such as wind, rainfall and presence of host cues, which were not addressed here. Additionally, the effects of long-term exposure to these conditions beyond 24-h is unclear. Further investigations into the effect of prolonged exposure to high and low temperature and humidity extremes on adult *Culicoides* activity and survival would be beneficial to assess their effects at the population level in northern Europe.

Several studies have suggested behavioral changes consequential to arbovirus infection in insect vectors which may influence their vectorial capacity. Specifically, aversion to UV light has been suggested in *Culicoides* infected with BTV (McDermott et al. 2015). A further study demonstrated a change in resting temperature preference of *C. sonorensis* held at 35 °C following vesicular stomatitis virus infection (Rozo-Lopez and Drolet 2024). It is therefore possible that arbovirus infection may alter the thermal tolerance of *Culicoides*, altering their activity and survival. Data underpinning current prediction models have so far focused on uninfected *Culicoides*. Further work should determine the potential effect of arbovirus infection on the flight activity of *Culicoides.* Understanding thermal biology of *Culicoides* may aid in understanding population dynamics, particularly distribution and abundance, providing further insight into the epidemiology of the arboviruses they transmit.

## Supplementary Material

tjag058_Supplementary_Data
