# Supplementary material for: Temperature and humidity limits for flight activity of field-collected Culicoides biting midges (Diptera: Ceratopogonidae) in the United Kingdom under defined laboratory conditions
Source: J Med Entomol. 2026 Apr 23;63(2):tjag058. doi: 10.1093/jme/tjag058 (PMC13105297; doi:10.1093/jme/tjag058)
Supplement: tjag058_Supplementary_Data [file tjag058_supplementary_data.zip › TableS2.docx]

**Table S2.** Coefficients in final generalised linear mixed model for *Culicoides* survival.

| parameter | estimate | 95% confidence interval | |
| --- | --- | --- | --- |
|  |  | lower | upper |
| intercept | 1.886 | 1.332 | 2.440 |
| meterological variables† |  |  |  |
| *T* | -0.634 | -0.825 | -0.443 |
| *H*_c_ | 0.977 | 0.487 | 1.467 |
| *H*_c_^2^ | -0.185 | -0.602 | 0.232 |
| *T*^2^*H* | -0.757 | -1.255 | -0.259 |
| season |  |  |  |
| Spring | 0.696 | -0.034 | 1.425 |
| Summer | 1.385 | 0.619 | 2.151 |
| Autumn | baseline | - | - |
| interactions |  |  |  |
| *H*: Spring | -0.687 | -1.331 | -0.043 |
| *H*: Summer | 0.137 | -0.530 | 0.805 |
| *H*^2^: Spring | -0.628 | -1.192 | -0.064 |
| *H*^2^: Summer | -1.118 | -1.698 | -0.538 |
| *T*^2^*H*: Spring | 0.301 | -0.324 | 0.925 |
| *T*^2^*H*:Summer | -0.567 | -1.230 | 0.097 |

† temperature (*T*) and relative humidity (*H*) were centred on their means and scaled by their standard deviations
