# Supplementary material for: Temperature and humidity limits for flight activity of field-collected Culicoides biting midges (Diptera: Ceratopogonidae) in the United Kingdom under defined laboratory conditions
Source: J Med Entomol. 2026 Apr 23;63(2):tjag058. doi: 10.1093/jme/tjag058 (PMC13105297; doi:10.1093/jme/tjag058)
Supplement: tjag058_Supplementary_Data [file tjag058_supplementary_data.zip › TableS1.docx]

**Table S1.** Coefficients in final generalised linear mixed model for *Culicoides* flight activity.

| parameter | estimate | 95% confidence interval | |
| --- | --- | --- | --- |
|  |  | lower | upper |
| intercept | -0.003 | -0.161 | 0.155 |
| meterological variables† |  |  |  |
| *T* | 0.155 | -0.023 | 0.334 |
| *T*^2^ | -0.868 | -1.023 | -0.713 |
| *H*_c_ | 0.138 | 0.028 | 0.248 |
| *H*_c_^2^ | -0.253 | -0.348 | -0.158 |
| *T*^2^*H* | 0.151 | 0.067 | 0.234 |
| *TH*^2^ | -0.100 | -0.238 | 0.039 |
| *T*^2^*H*^2^ | 0.253 | 0.114 | 0.392 |
| season |  |  |  |
| Spring | -0.141 | -0.314 | 0.032 |
| Summer | 0.078 | -0.102 | 0.257 |
| Autumn | baseline | - | - |
| interactions |  |  |  |
| *T*:Spring | -0.198 | -0.412 | 0.016 |
| *T*: Summer | 0.013 | -0.218 | 0.244 |
| *T*^2^: Spring | 0.481 | 0.318 | 0.644 |
| *T*^2^: Summer | -0.021 | -0.201 | 0.159 |
| *H*: Spring | 0.116 | -0.012 | 0.243 |
| *H*: Summer | -0.221 | -0.348 | -0.094 |
| *TH*^2^: Spring | 0.194 | 0.014 | 0.374 |
| *TH*^2^: Summer | -0.014 | -0.199 | 0.171 |
| *T*^2^*H*^2^: Spring | -0.383 | -0.539 | -0.226 |
| *T*^2^*H*^2^:Summer | -0.006 | -0.165 | 0.153 |

† temperature (*T*) and relative humidity (*H*) were centred on their means and scaled by their standard deviations
